# Comment on Peycheva et al. Trace Elements and Omega-3 Fatty Acids of Wild and Farmed Mussels (*Mytilus galloprovincialis*) Consumed in Bulgaria: Human Health Risks. *Int. J. Environ. Res. Public Health* 2021, *18*, 10023

**DOI:** 10.3390/ijerph20146393

**Published:** 2023-07-19

**Authors:** Chee Kong Yap, Meng Chuan Ong

**Affiliations:** 1 Department of Biology, Faculty of Science, Universiti Putra Malaysia, UPM Serdang 43400, Selangor, Malaysia; 2 Faculty of Science and Marine Environment, Universiti Malaysia Terengganu, Kuala Nerus 21030, Terengganu, Malaysia; 3 Ocean Pollution and Ecotoxicology (OPEC) Research Group, Universiti Malaysia Terengganu, Kuala Nerus 21030, Terengganu, Malaysia

First of all, the interesting paper by Peycheva et al. [1] was a good and informative literature source when I searched the internet. Thank you and congratulations.

Kindly, please allow me to comment on Peycheva et al.’s [1] assessment of the target cancer risk (TCR) of nickel (Ni). Peycheva et al. [1] determined the TCR of potentially toxic Ni in farmed mussels (*Mytilus galloprovincialis*) consumed in Bulgaria. Nonetheless, the presented TCR values of Ni were considered unacceptable because they were greater than the set limit of 10^−4^ [2]. This Ni risk assessment based on TCR values was questionable because of the following explanations. The Ni as 1.70 mg/kg bw/day was established for Ni subsulfide by USEPA [3] but not for elemental Ni in the food via the ingestion pathway. The USEPA’s Integrated Risk Information System does not clearly specify that 1.70 is an oral pathway for carcinogenic slope factor (CSF). However, Ni subsulfide (Ni_3_S_2_) has been shown convincingly to cause cancer after inhalation exposure [4], but no evidence has been demonstrated through the oral or ingestion pathway. This was the major reason why the Ni TCR was not included in the seafood samples [5].

Still, this Ni CSF has also been published in some high-ranked journals for fish samples through the ingestion pathway. For example, Liu et al. [6] reported the TCR values of Ni in the commercial marine organisms from Xiangshan Bay (China) while Adegbola et al. [7] also reported values greater than the set limit of 10^−4^ (cancer risks for Ni in the fish muscles of *Clarias gariepinus* and *Sarotherodon melanotheron* obtained from Ogun River, Nigeria).

Therefore, the use of CSF (1.70 mg/kg bw/day) to assess Ni TCR may cause many arguments and debates, thus rendering the Ni TCR values to be not convincing or acceptable. There is an urgent need to establish the CSF for elemental Ni in food, the CSF for the Ni oral pathway more specifically. As regular surveillance of Ni in commercial seafood is important, continual assessments for human health risks of Ni via ingestion can be a very potential research focus in the near future.
